# Supplementary material for: Cholesterol, PtdIns(4,5)P2, and Actin Regulate BK Channel Nanoscale Organization
Source: bioRxiv. 2026 Jun 2:2026.04.20.719652. Originally published 2026 Apr 22. Preprint. [Version 2] doi: 10.64898/2026.04.20.719652 (PMC13131582; doi:10.64898/2026.04.20.719652)
Supplement: 2 [file NIHPP2026.04.20.719652v2-supplement-2.pdf]

bioRxiv preprint doi: https://doi.org/10.64898/2026.04.20.719652; this version posted April 22, 2026. The copyright holder for this preprint (which was not certified by peer review) is the author/funder, who has granted bioRxiv a license to display the preprint in perpetuity. It is made available under aCC-BY-ND 4.0 International license.

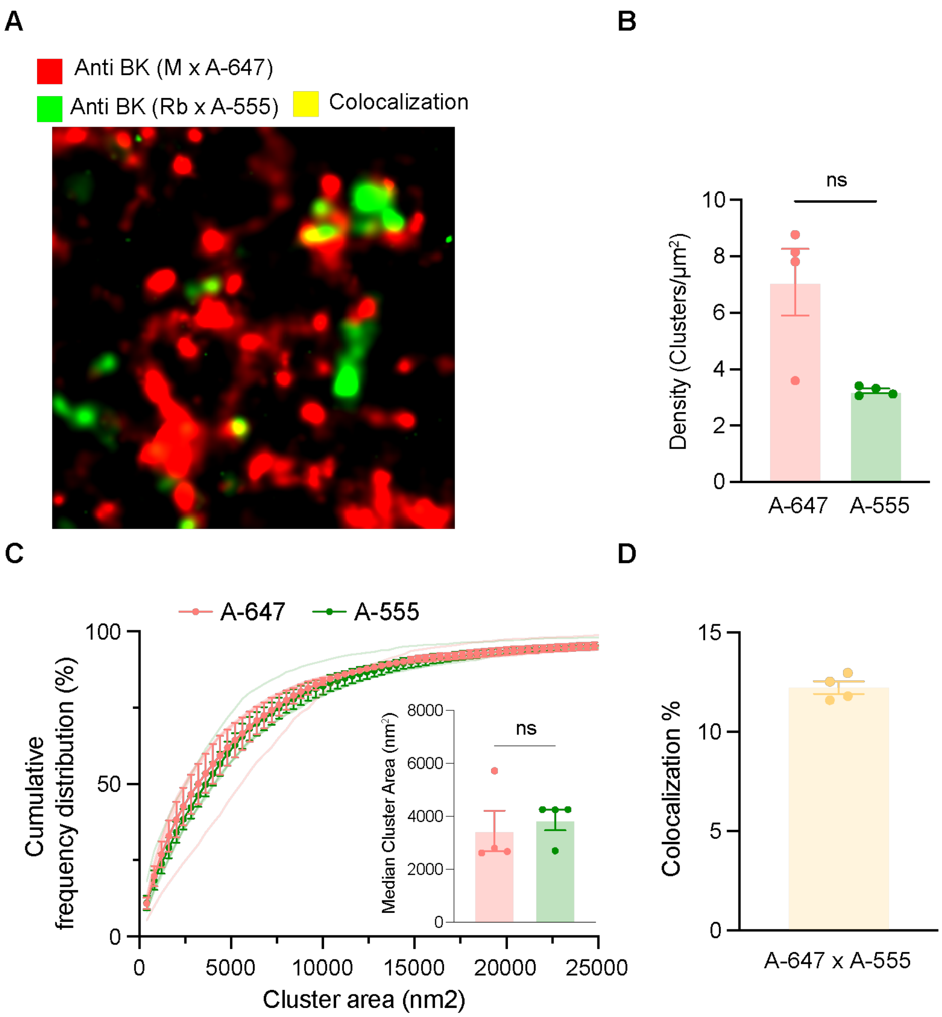


**Figure S1-1. Limited antibody colocalization supports single-site binding per BK tetramer. A.** Representative colocalization maps of the BK channel probed with two primary antibodies raised in mouse (red, Alexa-647) and rabbit (green, Alexa-555). **B.** Scatter plot showing BK cluster density (Clusters/µm$^2$) **C.** Relative cumulative frequency distribution of BK channels probed with two primary antibodies raised in mouse (red, Alexa-647) and rabbit (green, Alexa-555). Inset: scatter plot showing median cluster size (nm$^2$). **D.** Scatter plot showing colocalization between Alexa-647 and Alexa-555 signals. Data are from 4 cells shown as mean ± SEM.

bioRxiv preprint doi: https://doi.org/10.64898/2026.04.20.719652; this version posted April 22, 2026. The copyright holder for this preprint (which was not certified by peer review) is the author/funder, who has granted bioRxiv a license to display the preprint in perpetuity. It is made available under aCC-BY-NC-ND 4.0 International license.

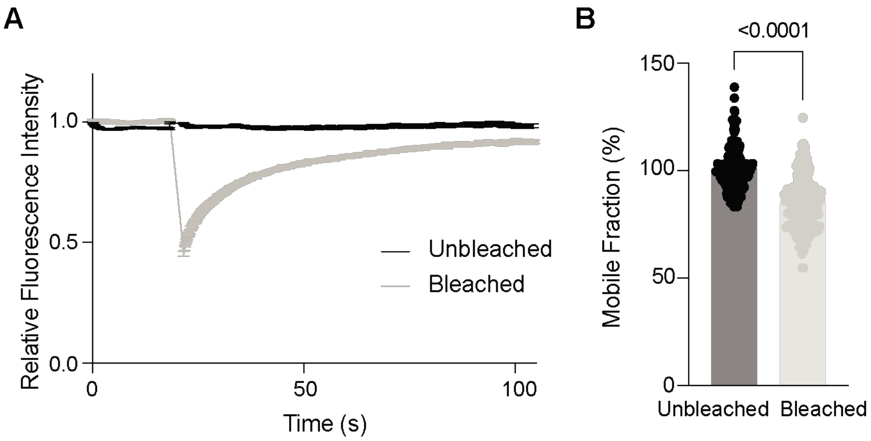


**Figure S1-2. Comparison of the change in fluorescence between bleached and unbleached areas**. **A**. Time course of fluorescence intensity from bleached (grey) and unbleaced (black) areas in tsA-201 cells transfected with fluorescently-tagged BK channels. **B**. Comparison of the percentage of change between the initial and final fluorescence fraction, representing the percentage mobile fraction. Statistical significance was tested using a two-tail t-test.

bioRxiv preprint doi: https://doi.org/10.64898/2026.04.20.719652; this version posted April 22, 2026. The copyright holder for this preprint (which was not certified by peer review) is the author/funder, who has granted bioRxiv a license to display the preprint in perpetuity. It is made available under a CC-BY-NC-ND 4.0 International license.

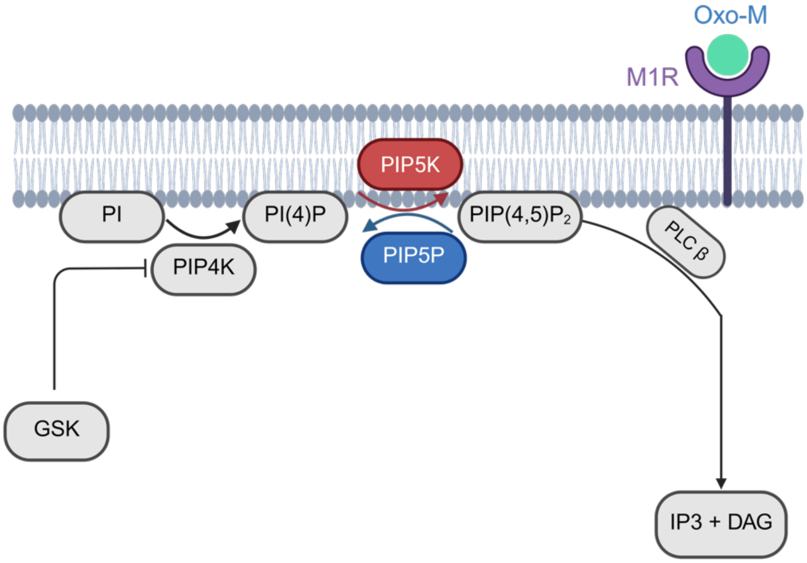


**Figure S2-1. Schematic illustrating experimental strategies used to manipulate PtdIns(4,5)$P_2$ abundance**. Chronic modulation was achieved by overexpressing PIP5K or PIP5P to increase or decrease PtdIns(4,5)$P_2$ synthesis, respectively. Acute depletion was induced by activating the M1 muscarinic receptor (M1R) with Oxotremorine-M (Oxo-M), which stimulates PLCβ-mediated hydrolysis of PtdIns(4,5)$P_2$, and inhibiting the PtdIns(4,5)$P_2$ synthesis pathway with GSK.
